# Slim-sugarcane: a lightweight and high-precision method for sugarcane node detection and edge deployment in natural environments

**DOI:** 10.3389/fpls.2025.1643967

**Published:** 2025-07-30

**Authors:** Lijiao Wei, Shuo Wang, Xinwei Liang, Dongjie Du, Xinyi Huang, Ming Li, Yuangang Hua, Weihua Huang, Zhenhui Zheng

**Affiliations:** ^1^ Agricultural Machinery Research Institute, Chinese Academy of Tropical Agricultural Sciences, Zhanjiang, China; ^2^ Key Laboratory of Agricultural Equipment for Tropical Crops, Ministry of Agriculture and Rural Affairs, Zhanjiang, China; ^3^ College of Engineering, South China Agricultural University, Guangzhou, China; ^4^ School of Information Technology & Engineering, Guangzhou College of Commerce, Guangdong, China

**Keywords:** sugarcane, detection, lightweight, edge deployment, TensorRT

## Abstract

Accurate detection of sugarcane nodes in complex field environments is a critical prerequisite for intelligent seed cutting and automated planting. However, existing detection methods often suffer from large model sizes and suboptimal performance, limiting their applicability on resource-constrained edge devices. To address these challenges, we propose Slim-Sugarcane, a lightweight and high-precision node detection framework optimized for real-time deployment in natural agricultural settings. Built upon YOLOv8, our model integrates GSConv, a hybrid convolution module combining group and spatial convolutions, to significantly reduce computational overhead while maintaining detection accuracy. We further introduce a Cross-Stage Local Network module featuring a single-stage aggregation strategy, which effectively minimizes structural redundancy and enhances feature representation. The proposed framework is optimized with TensorRT and deployed using FP16 quantization on the NVIDIA Jetson Orin NX platform to ensure real-time performance under limited hardware conditions. Experimental results demonstrate that Slim-Sugarcane achieves a precision of 0.922, recall of 0.802, and mean average precision of 0.852, with an inference latency of only 60.1 ms and a GPU memory footprint of 1434 MB. The proposed method exhibits superior accuracy and computational efficiency compared to existing approaches, offering a promising solution for precision agriculture and intelligent sugarcane cultivation.

## 1 Introduction

Sugarcane is an important sugar crop and a promising renewable energy resource. China has become the world’s third-largest producer of sugarcane, following Brazil and India. The annual sugarcane planting area exceeds 1.3 million hectares, with a yield of over 100 million tons (Li et al., 2024). As public awareness of food safety and quality continues to grow, traditional agricultural practices can no longer meet market demands. As a result, technological innovation in agriculture has become a key driver in the modernization of the sugarcane industry (Wan and Hu, 2023). At present, many field operations in sugarcane farming, such as seed cutting and planting, still rely heavily on manual labor. This leads to low operational efficiency and poor accuracy and uniformity in planting. With the advancement of smart agriculture, computer technologies have gradually been introduced into sugarcane cultivation and harvesting. These technologies help alleviate labor shortages and significantly improve productivity (Li, 2023; Dai et al., 2024b). However, current sugarcane nodes detection methods still suffer from low accuracy and poor efficiency. Such limitations hinder the progress of intelligent operations in sugarcane planting and harvesting. Therefore, developing a lightweight and accurate nodes detection system is a critical issue for achieving intelligent management in sugarcane fields.

At present, many researchers in China and abroad are engaged in the study of sugarcane node detection. These efforts can mainly be classified into two categories: traditional machine learning and deep learning methods. Moshashai et al. (2008) conducted a preliminary study on sugarcane node identification using a threshold segmentation method based on grayscale images. Chen et al. (2020) proposed a sugarcane nodes detection method based on the extreme points of a vertical projection function. Their method achieved recognition rates of 100%, 98%, and 95% for single, double, and triple nodes, respectively, with standard deviations of less than 1.1 mm, 1.7 mm, and 2.2 mm. Later, Chen et al. (2021b) introduced an algorithm that uses the local pixel sum at the minimum points of a vertical projection function. This method yielded recognition rates of 100% for single nodes and 98.5% for double nodes, with average processing times of 0.15 s and 0.21 s, respectively. Zhou et al. (2020) developed a machine vision-based sugarcane node cutting system. They computed gradient feature vectors and designed a positional search algorithm to locate nodes. The method achieved an average recognition rate of 93.00%, but only 17.00% for sugarcane with small internodal differences. Wang (2022) applied filtering, color space conversion, and image fusion to obtain binarized data. Then, using morphological segmentation and pixel distribution statistics, the method identified and located nodes. It achieved a 100% recognition rate for single-node sugarcane and over 80% for multi-node cases, with an execution time of 0.518 s. Shi et al. (2019) proposed a machine vision-based nodes detection method suitable for various sugarcane types. Their approach involved target extraction and correction, wavelet transform decomposition and reconstruction, and feature analysis. The recognition rate reached 92%. Huang et al. (2017) used a local mean-based method to segment the H component in the HVS color space and located nodes based on the maximum gray value. This method achieved a recognition rate of 90.77% with an average processing time of 0.481539 s. Zhang et al. (2016) used hyperspectral imaging to collect data through a spectrometer mounted above the image acquisition system. They extracted spectral bands representing nodes features to build a detection model. Lu et al. (2010) explored nodes feature extraction and detection using machine vision. They processed the S and H components of sugarcane images in the HSV color space and applied support vector machines to distinguish nodes and nodes. After clustering analysis, the average recognition rates for the number and location of nodes were 94.118% and 91.522%, respectively. Subsequently, Lu et al. (2012) analyzed grayscale image characteristics and used prior knowledge to statistically assess the gray values in RGB and HSV color spaces. Using gradient features, they identified nodes and found that the R component produced the best results, with a recognition accuracy of 88%. Although these traditional machine learning methods have achieved some progress in sugarcane node detection, they often rely on manually selected features. This limits their robustness and real-time performance under complex field conditions, reducing their practical applicability.

In recent years, with the rise and development of deep learning, it has gradually replaced traditional learning methods (Zheng and Xiong, 2021, 2023, Zheng et al., 2024a, 2024b, 2024c). Zheng et al. (2024a) optimized sugarcane nodes detection based on the YOLOv8 framework, achieving a mean average precision (mAP) of 0.974 and an inference time of 19.80 ms. Dai JX et al. (2024) improved the YOLOv5 network by introducing the CBAM attention mechanism and VarifocalNet, which increased the recognition accuracy of sugarcane nodes to 89.89%. He (2023) incorporated a multi-scale prediction structure and optimized bounding boxes using the K-means algorithm, raising the mAP of the improved YOLOv5 model to 93.8%. Zhao et al. (2023) further enhanced the YOLOv5 network, achieving a recognition accuracy of 97.1%. Chen (2022) proposed a hybrid detection model combining MobileNet and YOLOv4-t, which maintained detection accuracy while reducing model size. Li et al. (2022) improved the LeNet-5 architecture to enhance bud detection and positioning accuracy, reaching a recognition rate of 92% with a per-image detection time of 1.2 seconds. Tang (2021) optimized the YOLOv4 model by feeding valid feature layers directly into the enhanced feature extraction network and refining the path aggregation strategy. This reduced the recognition time per image to 6 ms, with accuracy reaching 98.68%. Li et al. (2019) simplified the YOLOv3 architecture by reducing the number of convolutional layers in residual blocks, resulting in a detection accuracy of 90.38% and an average detection time of 28.7 ms. Pan et al. (2024) combined MobileNetV3, U-Net, and an improved YOLOX to perform nodes contour segmentation and detection, achieving an MIoU of 91.68% and AP of 96.19%. Hu et al. (2025) proposed a lightweight network based on YOLOv8n-ghost for sugarcane nodes detection. By introducing Ghost modules to reduce redundancy, the model surpassed 90% AP and achieved real-time performance of nearly 30 frames per second after TensorRT acceleration. Dai et al. (2024a) developed an improved YOLOv5s-KCV model integrating K-means clustering, the CBAM module, and the VarifocalNet mechanism. The model achieved a precision of 89.89%, a recall of 89.95%, and a mAP of 92.16%. Xie et al. (2024) introduced the G-YOLOv5s-SS lightweight model, which integrated Ghost modules and the SimAM mechanism. This approach achieved a recognition accuracy of 97.6%. Wen et al. (2023) proposed an improved sugarcane nodes detection model based on YOLOv7. It incorporated the SimAM attention mechanism, deformable convolutions, and WIoU loss to improve both accuracy and robustness. The resulting model achieved a mAP of 94.53% and an F1 score of 92.41%. Yu et al. (2023) proposed a lightweight MobileNetv2-YOLOv5s model for real-time detection of sugarcane nodes in complex natural environments. By replacing the YOLOv5s backbone with MobileNet, model complexity was reduced by 40%, with only a 0.8% drop in AP and a detection speed of 4.4 ms. Wang et al. (2022) enhanced YOLOv4-Tiny by integrating an SPP module and 1×1 convolution layers, improving both localization accuracy and speed. Their model achieved a mAP of 99.11%, a precision of 97.07%, and a frame rate of 30 fps. Zhou et al. (2022) proposed an algorithm combining YOLOv3 and traditional computer vision techniques. They used affine transformation for posture correction, YOLOv3 for initial detection, and gradient operators with local thresholding for precise localization. The method achieved a recognition accuracy of 99.68% and a recall rate of 100%. Zhu et al. (2022) introduced an improved YOLOv4-based method for nodes detection, reaching an AP of 94.4%. Chen et al. (2021a) developed a YOLOv4-based detection algorithm that achieved 95.17% AP and a speed of 69 frames per second. Despite the progress in improving model accuracy, most of these studies rely on high-performance computing equipment. This makes them unsuitable for deployment in practical agricultural scenarios, where low cost and high efficiency are critical.

Unlike previous studies, this paper proposes a high-precision and lightweight method for sugarcane nodes detection, aiming to address key challenges of large model size, limited deployability, and insufficient performance in existing approaches. By improving critical components of the detection system, we ultimately present an end-to-end, low-cost solution for sugarcane nodes recognition. To the best of our knowledge, this is among the first studies to achieve robust sugarcane node detection with real-time performance on edge devices under complex natural environments. The main contributions are summarized as follows:

(1) We propose a lightweight sugarcane node detection model, Slim-Sugarcane, which integrates GSConv to replace standard convolution and introduces a Cross-Stage Local Network module to reduce model redundancy and computational complexity.
(2) The model is quantized and accelerated using the TensorRT framework, and an FP16-based deployment scheme is proposed. This approach enhances deployment performance and response speed while maintaining detection accuracy. Furthermore, comparative evaluations are conducted across different deployment platforms to assess performance variations.
(3) The optimized model is deployed on the Nvidia Orin NX edge device, and field experiments are conducted in a sugarcane plantation to evaluate the model’s performance and resource consumption under real-world deployment conditions.

## 2 Materials and methods

### 2.1 Image acquisition method

This study utilized a dataset collected from a sugarcane field at the Agricultural Mechanization Research Institute of the Chinese Academy of Tropical Agricultural Sciences, located in Zhanjiang, Guangdong Province, China (21°10′N, 110°16′E). Image acquisition experiments were conducted in November 2023 and October 2024. During the experiments, RGB images were captured using an iPhone 13 and a HUAWEI Mate 60 Pro under automatic camera settings. All images were recorded at a resolution of 4032×3024 pixels and saved in JPG format. A total of 550 images were collected, including 314 images taken on a mechanical cutting machine and 236 images captured in natural field conditions after machine planting. Each image contained 3 to 5 sugarcane nodes, resulting in approximately 2,100 annotated nodes across the dataset. The combination of controlled and real-field conditions ensures variability in lighting, occlusion, and background complexity, contributing to better generalization during model training.

### 2.2 Image data preprocessing

The quality of the dataset plays a decisive role in the accuracy of the trained recognition model and its performance in practical applications. To ensure data clarity and representativeness, 500 sugarcane images were carefully selected. At the same time, to improve the reliability of model training and validation, these images were divided into training, validation, and test sets in a ratio of 7:1.5:1.5. Specifically, 350 images were used for training, 75 for validation, and the remaining 75 for testing. The detailed dataset split is shown in **Table 1**.In this study, the widely used annotation tool in the object detection field, LabelImg (https://github.com/tzutalin/labelImg), was employed to label the sugarcane dataset. The generated label files contain class information for each sugarcane nodes as well as its normalized position within the images.

**Table 1:** Details of the dataset.

Scene Dataset	Training set	Validation set	Test set	Number of nodes
Indoor	210	45	45	1196
Outdoor	140	30	30	929

## 3 Methodology

### 3.1 Lightweight sugarcane nodes detection network

#### 3.1.1 Yolov8

**Figure 1:**
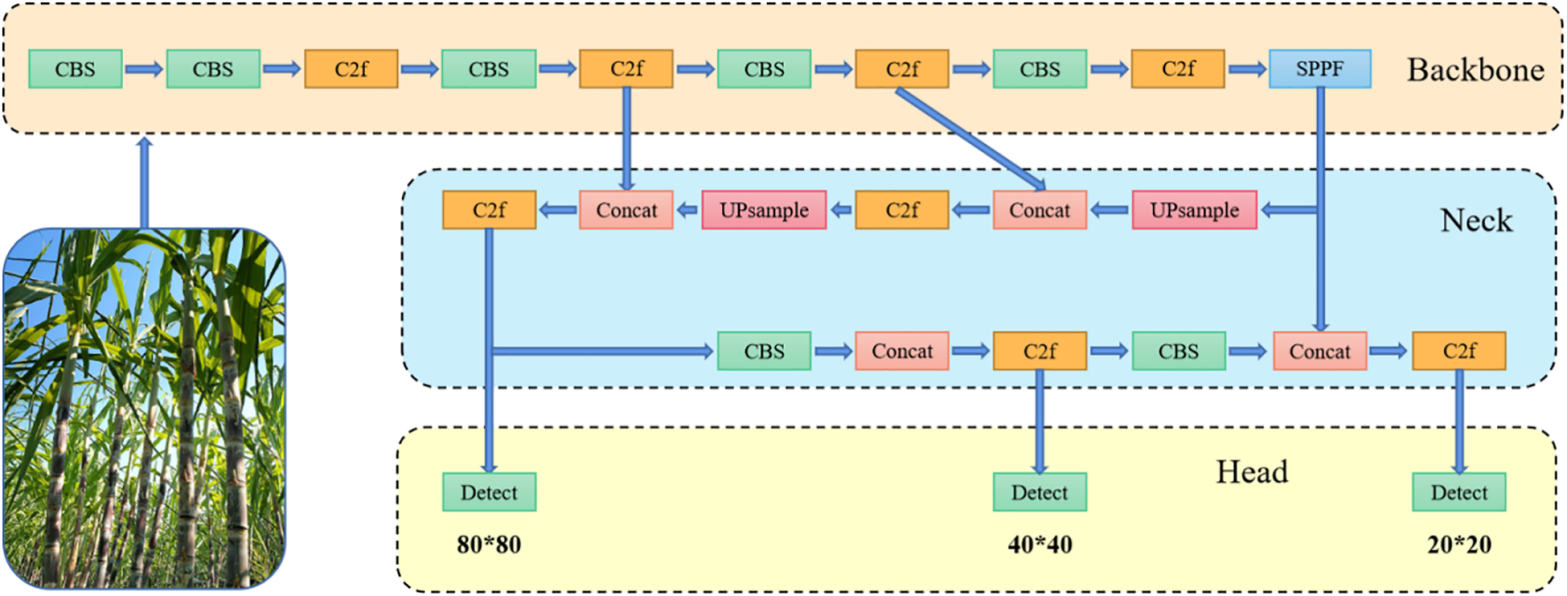
YOLOv8 network architecture diagram.

Accurate sugarcane node identification is essential for intelligent planting and harvesting, especially under variable outdoor conditions. YOLOv8 has been widely adopted in agricultural detection tasks due to its strong balance between accuracy and speed. The model features a streamlined architecture with a lightweight backbone composed of Conv, C2f, and SPPF modules. It employs a Feature Pyramid Network (FPN) and Path Aggregation Network (PAN) in the neck to fuse multi-scale features, and utilizes a decoupled detection head for classification and localization, as illustrated in **Figure 1**. This design significantly enhances detection performance while ensuring efficient computation. Moreover, YOLOv8 supports deployment on edge devices without requiring high-performance GPUs, making it suitable for practical agricultural applications. Given these advantages, YOLOv8-l was selected as the baseline framework for this study. Nonetheless, to meet the strict constraints of edge deployment in real-world agricultural environments, further architectural optimization is necessary to reduce redundancy, minimize computational cost, and enhance robustness.

#### 3.1.2 Slim-sugarcane

**Figure 2:**
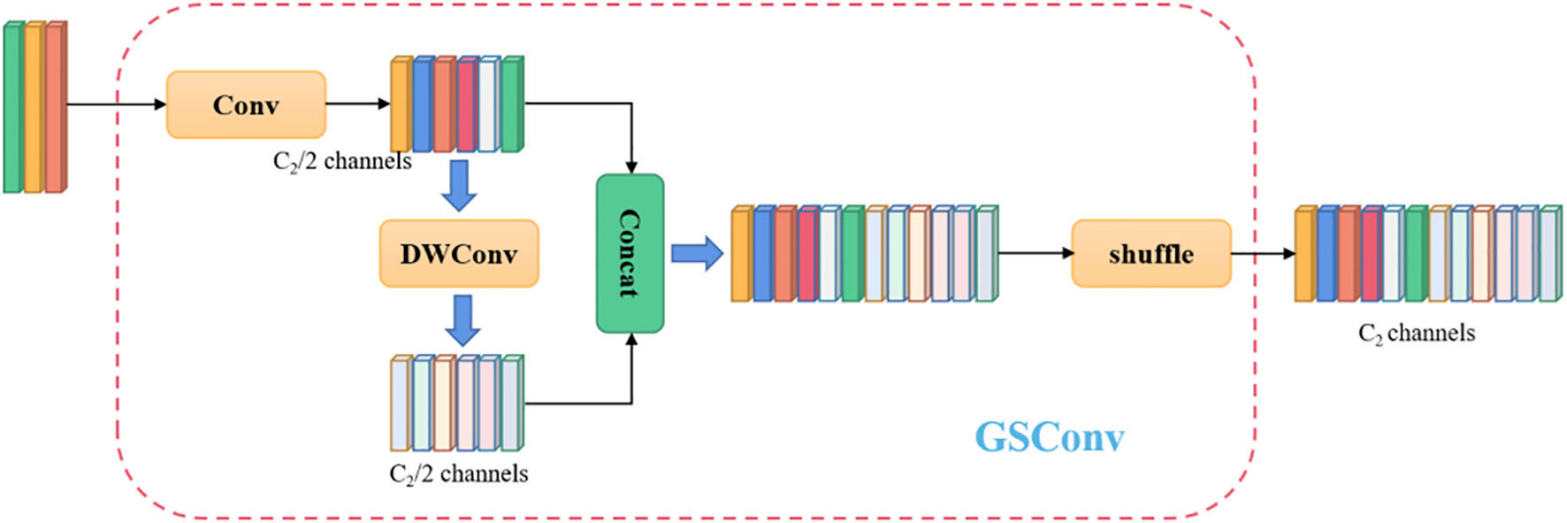
The structure of the GSConv.

**Figure 3:**
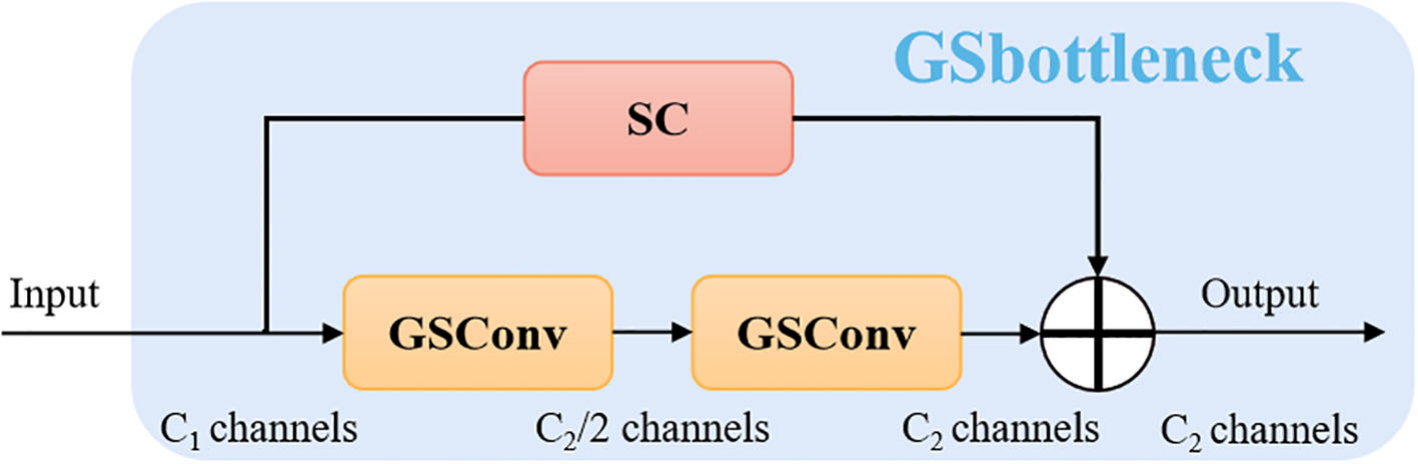
The structures of the GSbottleneck module.

To address the limitations of the traditional YOLOv8 model—such as redundant convolutions, excessive parameter count, and suboptimal adaptability to edge hardware—this study proposes a novel detection model named Slim-Sugarcane. The model is designed to improve both the accuracy and efficiency of node detection while meeting the deployment requirements of edge computing environments, thereby facilitating its application in real-world agricultural scenarios. Considering the limited computational resources in sugarcane fields and the practical constraints of deploying models on edge devices, we constructed a simple and efficient neck module, introducing grouped shuffle convolution and a custom-designed Slim-Neck architecture. A Slim-Neck-based feature fusion module is proposed to better aggregate multi-scale information. To accelerate inference speed, the input images are passed through a series of transformations in the backbone network, gradually projecting spatial information into the channel dimension. As the width and height of spatial features decrease, the number of feature channels correspondingly increases. However, this process inevitably leads to the loss of some semantic information. While dense convolutions help preserve these connections, sparse convolutions may disrupt them. To retain feature diversity while improving computational efficiency, we adopt a channel shuffling strategy to allow features generated by standard convolution (SC) to permeate through those generated by depthwise separable convolution (DSC). This results in the implementation of a lightweight GSConv module, as shown in **Figure 2**, which replaces the standard convolutional block CBS. On top of GSConv, we introduce a GSBottleneck module, a lightweight bottleneck structure that further reduces computational cost while maintaining detection accuracy, as illustrated in **Figure 3**.

**Figure 4:**
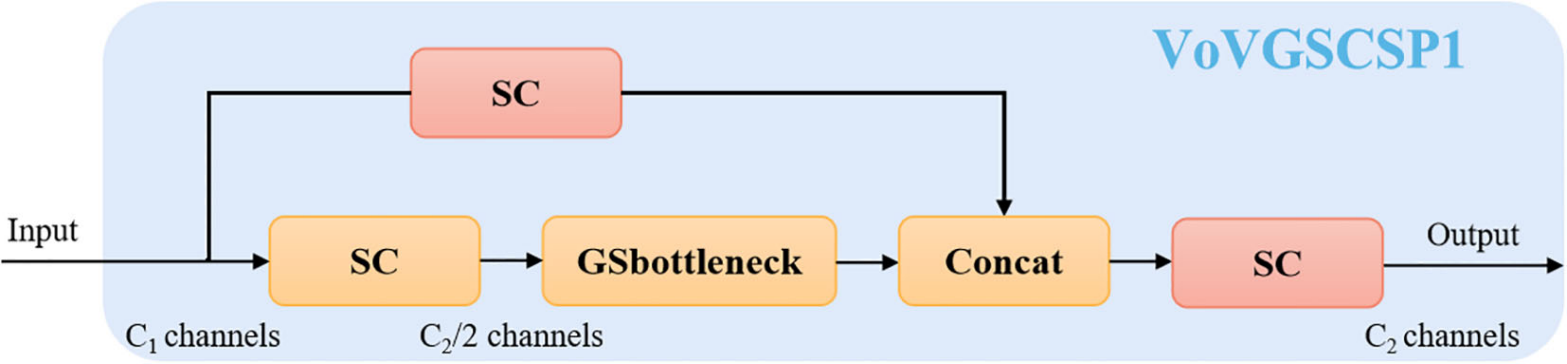
The structures of the VoV-GSCSP module.

The C2f module in YOLOv8 consists of multiple standard convolutions and bottleneck structures, extracting multi-scale features through residual connections. However, this design introduces additional parameters and computational overhead, increasing reliance on computing resources. Moreover, as the network depth grows, the C2f module’s ability to represent complex semantic information gradually declines, making it less effective in handling varying object scales and complex background interference in sugarcane images. To address these issues, we adopt a one-shot aggregation strategy to design a cross-stage partial network module, referred to as VoV-GSCSP, illustrated in **Figure 4**. This approach reduces both computational and structural complexity while maintaining sufficient detection accuracy.

Therefore, this study replaces the standard CBS convolution blocks and the original C2f modules in the neck of the model with the GSConv convolution module and the VoV-GSCSP module for feature processing. This substitution effectively reduces computational load and accelerates inference speed. The improved Slim-Sugarcane model enhances detection accuracy in complex environments and is well-suited for deployment in edge computing scenarios, making it more applicable to intelligent sugarcane farming.

#### 3.1.3 TensorRT-based quantization acceleration

**Figure 5:**
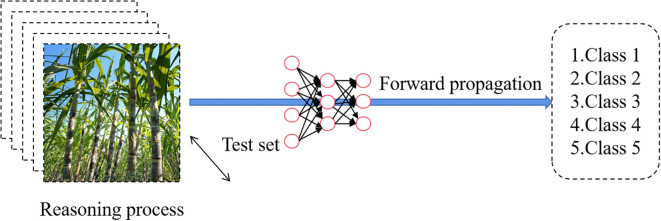
Reasoning process.

Existing detection models typically feature large-scale architectures and demand significant computational resources, posing challenges for real-time execution on embedded devices. To meet the dual requirements of real-time performance and low power consumption for sugarcane node detection in field operations, it is essential to optimize the trained model for deployment. In this study, we adopted TensorRT, a deep learning inference optimizer developed by NVIDIA. TensorRT enhances deployment performance and response speed of the sugarcane node detection model in real agricultural environments by optimizing computational workflows and accelerating the inference process. The inference workflow is illustrated in **Figure 5**.

**Figure 6:**
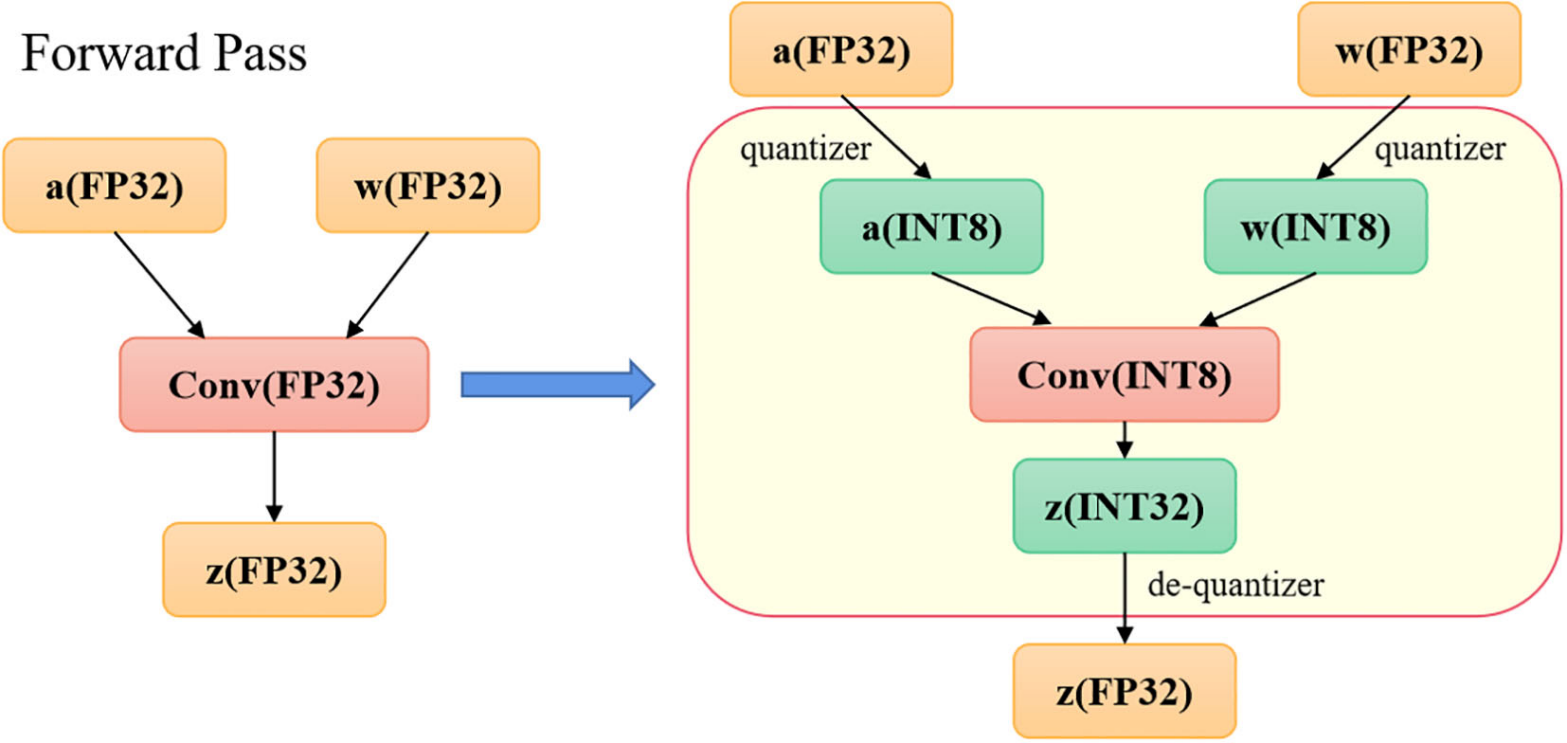
Quantization process.

TensorRT provides five primary optimization techniques, including precision calibration for weights and activations, layer and tensor fusion, automatic kernel selection, dynamic tensor memory allocation, and multi-stream execution. In this study, we focus on precision calibration for weights and activations, using Int8 quantization as an example. In deep learning frameworks, neural network training typically relies on 32-bit floating-point (FP32) computations. However, during the inference phase, as backpropagation is not required, precision can be reduced appropriately. Int8 quantization converts FP32 parameters into 8-bit integers, which significantly compresses model size, reduces memory usage and power consumption, and improves inference speed and overall performance—thereby lowering the operational cost of the model. The Int8 quantization method linearly maps activation values and weights from FP32 to INT8. Convolution operations are then performed using INT8 weights and activations, producing INT32 outputs, which are subsequently re-quantized back to INT8 and used as inputs for the next layer. For the final layer of the network, a dequantization step converts the INT8 outputs back to FP32. The complete workflow is illustrated in **Figure 6**.

Through the aforementioned process, the improved model achieves enhanced real-time performance while maintaining detection accuracy. By integrating the proposed Slim-Sugarcane network, the system can accurately detect sugarcane nodes even under complex natural conditions. This effectively meets the practical demands of intelligent sugarcane cultivation and harvesting.

## 4 Experimental results and analysis

### 4.1 Experimental setup and parameters

To ensure a fair evaluation of algorithm performance, all models were trained and tested under a unified experimental platform with consistent hyperparameter settings. The experimental environment consisted of a 13th-generation Intel Core i9-13900K processor (3 GHz, 24 physical cores, 32 threads), an NVIDIA GeForce RTX 4090 GPU, Ubuntu 18.04 operating system, CUDA version 11.1.74, OpenCV 4.8.0, and the PyTorch version 2.0.1.

(1) Parameter settings: The YOLOv6-l and YOLOv8-l official pretrained model was selected as the initial model. The image size of the dataset was set to 640×640 pixels, and the maximum number of training epochs (Max_epoch) was set to 500. The batch size (Batch_size) was configured to 24, with an initial learning rate of 0.01, a momentum factor of 0.90, and a weight decay coefficient of 0.0005.
(2) Training strategy: During the training process, the K-means clustering algorithm was employed to accurately determine the optimal aspect ratios of anchor boxes. To enhance the model’s generalization ability and robustness, various image augmentation techniques were applied. Firstly, the Mosaic data augmentation was used to increase the diversity of training samples and the complexity of backgrounds. Secondly, the Mixup method was adopted to generate new training samples by linearly interpolating both images and labels. Additionally, the Exponential Moving Average (EMA) technique was introduced to smooth the model parameters and improve stability. In terms of color space augmentation, HSV color space enhancement was applied by adjusting image saturation and brightness to accommodate different lighting and color variations. Finally, horizontal flipping was performed to enhance the model’s symmetry recognition capability. To prevent overfitting, an EarlyStopping mechanism was implemented to automatically terminate training when the validation loss stopped improving.

### 4.2 Evaluation metrics

To comprehensively evaluate the performance of the model, this study employs precision, recall, mean Average Precision (mAP), F1 score, single-frame inference time, and model size as the evaluation metrics for the sugarcane node detection network. The calculation formulas are as follows (Equations 1–4):


(1) $$P=\frac{T_{p}}{T_{p}+F_{p}}$$

(2) $$R=\frac{T_{p}}{T_{p}+F_{N}}$$

(3) $$mAP=\sum_{i=1}^{C}\frac{AP_{i}}{C}$$

(4) $$F1=\frac{2\times P\times R}{P+R}$$

Here, TP denotes true positives, where the actual samples are positive and predicted as positive; FP denotes false positives, where the actual samples are negative but predicted as positive; FN denotes false negatives, where the actual samples are positive but predicted as negative, representing the number of undetected sugarcane nodes. P and R stand for precision and recall, respectively, which are crucial metrics for evaluating the performance of detection models. AP (Average Precision) corresponds to the area under the precision-recall curve, reflecting the overall model performance. Inference time refers to the time required to detect sugarcane nodes in a single image, serving as a measure of the model’s efficiency. Model size indicates the scale of the model, with the number of network parameters playing a key role in practical deployment, significantly impacting the model’s running speed and overall performance.

### 4.3 Field experiments on sugarcane node detection

#### 4.3.1 Comparative experiments of different network models

To verify the effectiveness of the proposed method, a comprehensive comparison was conducted between the Slim-Sugarcane model and several state-of-the-art models including YOLOv6, YOLOv8, and its variants (YOLOv8-ConvNeXtV2, YOLOv8-LSKNet, YOLOv8-FasterNet, YOLOv8-C2f-DBB) on the test set. **Table 2** presents the testing results of our model alongside these advanced models for the sugarcane node detection task. The evaluation metrics used include precision, recall, F1-score, mAP, inference time, and model size.

**Table 2:** Comparison of detection performance of different networks.

Model	P	R	F1	mAP	Inference time	Model size
YOLOv6	0.87	0.77	0.82	0.81	19.6	52.7
YOLOv8	0.90	0.78	0.84	0.86	18.2	87.6
YOLOv8-convnextv2	0.77	0.72	0.74	0.75	13.6	55.7
YOLOv8-LSKNet	0.84	0.76	0.80	0.79	12.4	56.2
YOLOv8-Fasternet	0.89	0.77	0.83	0.82	7.8	52.7
YOLOv8-c2f-dbb	0.91	0.77	0.83	0.84	10.9	163.7
Slim-Sugarcane	0.91	0.79	0.85	0.86	12.2	70.9

As shown in **Table 2**, the Slim-Sugarcane network achieved a precision of 0.91, recall of 0.79, F1-score of 0.85, mAP of 0.86, inference time of 12.2 ms per image, and a model size of 70.9 MB on the test set. Compared to the unmodified YOLOv6 and YOLOv8 models, the F1-score of Slim-Sugarcane improved by 3.0% and 1.0%, respectively. Moreover, the mAP increased by 5.0% over YOLOv6, while the per-frame inference time was reduced by 6.0 ms and 7.4 ms, respectively. The model size was also reduced by 16.7 MB compared to YOLOv8.When compared with other YOLOv8 variants, the proposed model improved detection precision by 2.0% to 14.0% and F1-score by 1.0% to 11.0%. These results indicate that the Slim-Sugarcane network is more effective in detecting targets, with reduced false negatives and false positives. The results demonstrate that the proposed sugarcane node detection network offers significant advantages in both detection accuracy and inference speed. These improvements stem from the adoption of GSConv, which replaces conventional standard convolutions (CBS), enabling a more efficient combination of grouped and spatial convolutions to reduce computational cost while maintaining strong detection performance. Additionally, the model incorporates a single-stage aggregation strategy to construct a cross-stage local perception module (GSCSP), which further reduces computational complexity and structural redundancy without sacrificing accuracy, thereby accelerating inference speed. Furthermore, the lightweight nature of the Slim-Sugarcane model enhances its deployability on edge devices.

**Figure 7:**
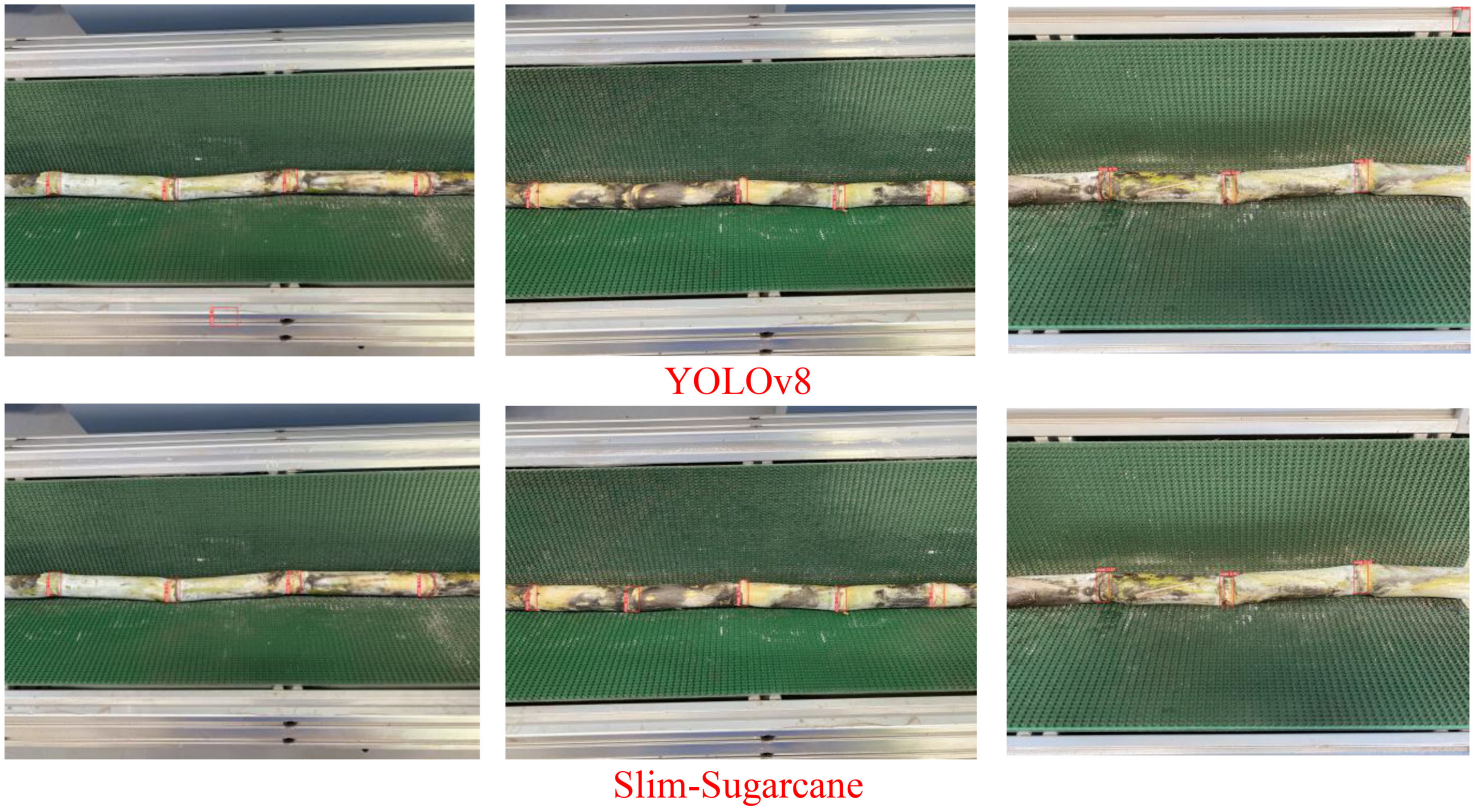
Comparison of two algorithms in an indoor seed cutter operating environment.

**Figure 8:**
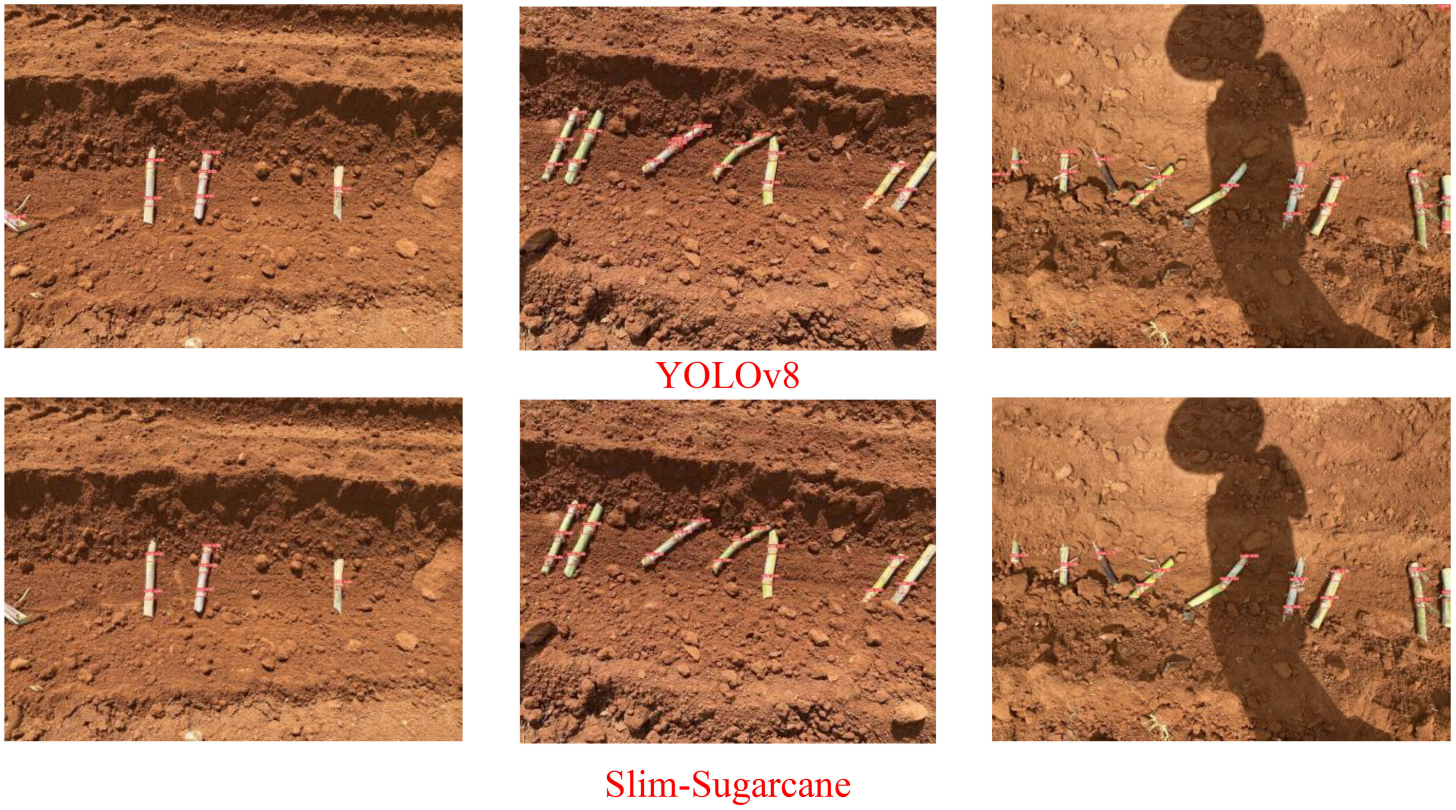
Comparison of two algorithms in a field grower operating environment.

As illustrated in **Figure 7**, under indoor conditions with the seed-cutting machine in operation, both the Slim-Sugarcane and YOLOv8 networks are capable of accurately detecting sugarcane nodes in the images. Compared to YOLOv8, the Slim-Sugarcane network effectively reduces the occurrence of missed and false positives. In contrast, during actual mechanized planting operations in outdoor sugarcane fields, environmental factors such as mud occlusion, airborne dust, and variable natural lighting frequently degrade image quality, introducing substantial challenges for robust node detection. These real-world disturbances can lead to blurry visuals, partial occlusions, and inconsistent contrast, which may significantly affect model performance. **Figure 8** presents the detection results of the Slim-Sugarcane network under such challenging field conditions. The Slim-Sugarcane network maintains high detection accuracy, effectively avoiding missed detections, false positives, and redundant bounding boxes that are more commonly observed in the YOLOv8 results. These findings highlight the model’s robustness and generalization capability in complex, variable outdoor agricultural environments, reinforcing its suitability for real-time deployment in practical sugarcane production scenarios.

#### 4.3.2 Comparative experiments on different quantization methods

To enable real-time deployment of the proposed network on edge devices with reduced computational overhead and shorter inference latency, we applied TensorRT-based quantization acceleration to enhance the deployment efficiency and response speed of the sugarcane node detection model. In this study, we evaluated and compared three quantization precision configurations: FP32, FP16, and INT8. By comparing the inference speed and detection accuracy under these three weight precision settings, we aimed to assess the model’s effectiveness in practical edge computing scenarios.

As shown in **Table 3**, the FP16 model significantly reduces the inference time per image from 12.2 ms to 8.4 ms, achieving a 1.45× increase in inference speed while maintaining nearly the same accuracy and recall as the original FP32 model. This enhancement markedly improves the system’s real-time processing capability. Although the INT8 quantized model demonstrates faster inference, it suffers from a considerable loss in detection accuracy, rendering it unsuitable for the high-precision requirements of sugarcane node detection in field operations. In contrast, the FP16 model preserves the robustness and stability of the original model without requiring complex quantization-aware training, and it offers excellent hardware compatibility. Experimental results confirm that the TensorRT-FP16 deployment scheme provides millisecond-level response times suitable for real-time field applications. Furthermore, its high recognition accuracy effectively addresses challenges posed by outdoor environments, minimizing missed and false detections. This solution offers strong support for the intelligent automation of sugarcane planting and harvesting operations.

**Table 3:** Comparison of model performance under different quantization precisions.

Model	P	R	Preprocess	Inference	Postprocess
FP32	0.916	0.794	0.3ms	8.9ms	3.0ms
FP16	0.916	0.794	0.7ms	4.7ms	3.0ms
INT8	0.393	0.190	0.8ms	4.1ms	4.2ms

#### 4.3.3 Field experiment

In actual sugarcane fields, the implementation of real-time node recognition and processing faces challenges due to limited device resources and unstable network conditions. To address these constraints, deploying the model on edge computing devices enables end-to-end sugarcane node recognition and localization, ensuring accurate detection even under resource-limited conditions and thereby enhancing the overall system reliability. To evaluate detection performance across different platforms and deployment schemes, this study conducts a comparative analysis of the Slim-Sugarcane model and its TensorRT-FP16 optimized version on both the RTX 4090 and NVIDIA Orin NX platforms, aiming to assess the model’s effectiveness in real-world sugarcane field scenarios. All inference tests were conducted using an input resolution of 640×640 pixels and a batch size of 1. The deployment on the NVIDIA Orin NX edge device was implemented using TensorRT v8.5.2.2, CUDA v11.4, cuDNN v8.6.0.166, and JetPack v5.1.

As shown in **Table 4**, the Slim-Sugarcane model achieved a precision of 0.916, a recall of 0.794, and a mAP of 0.860 on the RTX 4090 platform. When deployed on the NVIDIA Orin NX without optimization, the inference time increased to 151.9 ms, reflecting the performance impact under limited computational resources. After applying TensorRT-FP16 acceleration on the Orin NX, the inference time was reduced to 60.1 ms, and the preprocessing and postprocessing times also decreased. In terms of GPU memory usage, the optimized model occupied 1434 MB, compared to 1536 MB for the unoptimized version. The precision and recall remained consistent after optimization, indicating that inference speed improvements were achieved without compromising detection accuracy. These results demonstrate that the Slim-Sugarcane model can operate within the hardware constraints of the Orin NX device and maintain stable performance under field conditions.

**Table 4:** Comparison of model performance before and after deployment.

Data Metrics	Slim-Sugarcane-TensorRT-FP16 on RTX 4090	Slim-Sugarcane on ORIN	Slim-Sugarcane-TensorRT-FP16 on ORIN
P	0.916	0.922	0.922
R	0.794	0.802	0.802
mAP	0.86	0.852	0.852
Inference time/ms	4.7	151.9	60.1
Preprocessing time/ms	0.7	2.8	1.2
Postprocessing time/ms	3.0	6.2	3.4
Gpu memory/MB	990	1536	1434

## 5 Discussion

To address the key issues of large model size, deployment difficulty, and limited performance in existing sugarcane node detection methods, this paper proposes a lightweight sugarcane node detection and localization system named Slim-Sugarcane. By replacing the standard CBS convolution blocks and the original neck’s C2f module in the YOLOv8 architecture with GSConv convolution modules and VoV-GSCSP modules, the model reduces computational complexity and accelerates inference speed. Furthermore, an FP16 precision TensorRT acceleration deployment strategy is employed to realize end-to-end deployment on the Nvidia Orin NX edge device. The system’s performance, including inference speed and resource consumption, is evaluated in real-world sugarcane field environments.

Existing methods, such as those by Chen et al. (2020); Zhou et al. (2020); Huang et al. (2017), and Lu et al. (2010), mainly rely on manually selected and designed features. Although these approaches demonstrate certain recognition capabilities in specific scenarios, they generally suffer from insufficient real-time performance and poor robustness to environmental changes. In recent years, deep learning-based methods like those proposed by Zheng et al. (2024); Hu et al. (2025); Yu et al. (2023), and Zhu et al. (2022) have significantly improved detection efficiency. However, these models typically require high-performance hardware for operation, which does not meet the low-cost and high-efficiency demands of practical agricultural applications, and their performance remains vulnerable to environmental factors. In contrast, the Slim-Sugarcane network proposed in this paper not only improves recognition accuracy but also accelerates detection speed. Moreover, it significantly reduces missed detections and duplicate detections, even under challenging conditions where image quality is degraded by complex field environments.

The innovation of this study lies in achieving both lightweight and efficient detection, with deployment on a low-cost Nvidia Orin NX edge device accelerated by TensorRT. Field experiments validate the model’s robustness under complex environmental conditions. Compared to traditional cloud-based solutions, this approach significantly reduces computational resource consumption and latency while maintaining detection accuracy. Although the system performs well in terms of precision, speed, and efficiency, the experiments also reveal certain limitations. Firstly, when dense sugarcane leaves occlude the node areas, the model may mistakenly identify leaf edges as node boundaries due to their similar texture features. Secondly, under strong midday sunlight, intense reflections on the node surface can create bright spots that interfere with feature extraction, causing the model to generate multiple overlapping detection boxes at the same node location. Future work will focus on addressing these issues and continuously optimizing the detection algorithm to promote its practical application in intelligent sugarcane cutting and precision planting equipment.

## 6 Conclusions

This study proposes the Slim-Sugarcane lightweight sugarcane node detection network, an improved version based on YOLOv8. The model integrates TensorRT acceleration and is ultimately deployed on the Nvidia Orin NX edge computing device. The main conclusions are summarized as follows:

(1) This paper presents the Slim-Sugarcane model, which effectively reduces computational overhead and improves inference speed by introducing modules such as SlimNeck and GSConv. Compared with the original model, Slim-Sugarcane maintains the same mAP while reducing the inference time per frame by 6 ms and the model size by 16.7 MB. Comparative experiments with YOLOv6, YOLOv8, and its variants demonstrate that the Slim-Sugarcane model significantly enhances both the accuracy and efficiency of sugarcane node detection tasks.
(2) By integrating TensorRT acceleration, this paper proposes an FP16 quantization-based deployment optimization scheme. Without compromising the original model’s accuracy, the total processing time per frame is reduced from 12.2 ms to 8.4 ms, achieving a speedup of approximately 1.45×. Experimental results demonstrate that the TensorRT-FP16 solution achieves a balanced trade-off between accuracy and speed while offering strong adaptability and robustness, making it well-suited for fast sugarcane node detection tasks in complex field environments.
(3) The Slim-Sugarcane model was deployed on the Nvidia Orin NX edge device, and field experiments were conducted in an actual sugarcane plantation to evaluate the model’s performance and resource consumption under real-world conditions. The study further compares the runtime performance of different model versions on both high-performance and edge computing platforms. Experimental results show that the Slim-Sugarcane-TensorRT-FP16 model achieves a mean Average Precision of 85.2% on the edge device, with an inference time of approximately 60.1 ms, a total preprocessing and postprocessing time of 4.6 ms, and GPU memory usage of 1434 MB. These results indicate that the proposed model maintains stable accuracy and fast response under the resource constraints of edge computing, demonstrating its practicality and reliability for real-time sugarcane node detection in complex field environments.

## Data Availability

The raw data supporting the conclusions of this article will be made available by the authors, without undue reservation.
